# Role of D3 dopamine receptors in modulating neuroanatomical changes in response to antipsychotic administration

**DOI:** 10.1038/s41598-019-43955-4

**Published:** 2019-05-24

**Authors:** Elisa Guma, Jill Rocchetti, Gabriel A. Devenyi, Arnaud Tanti, Axel P. Mathieu, Jason P. Lerch, Guillaume Elgbeili, Blandine Courcot, Naguib Mechawar, M. Mallar Chakravarty, Bruno Giros

**Affiliations:** 1 Integrated Program in Neuroscience, McGill University, Montreal, Quebec H3A2B4 Canada; 2 Cerebral Imaging Center, Douglas Mental Health University Institute, Montreal, Quebec H3H1R3 Canada; 3 McGill Group for Suicide Studies, Department of Psychiatry, McGill University, Douglas Mental Health University Institute, Montreal, QC Canada; 4 Department of Psychiatry, McGill University, Montreal, Quebec H3A1A1 Canada; 5 Department of Biological and Biomedical Engineering, McGill University, Montreal, Quebec H3A2B4 Canada; 6 Mouse Imaging Centre, The Hospital for Sick Children, Toronto, Ontario M5T3H7 Canada; 7 Department of Neurosciences and Mental Health, The Hospital for Sick Children, Toronto, Ontario M5G1X8 Canada; 8 Department of Medical Biophysics, University of Toronto, Toronto, Ontario M5G1L7 Canada; 9 Wellcome Centre for Integrative Neuroimaging, University of Oxford, Oxford, UK; 10 Sorbonne University, Neuroscience Paris Seine, CNRS UMR 8246, INSERM U 1130, UPMC Univ Paris 06, UM119, 75005 Paris France

**Keywords:** Schizophrenia, Schizophrenia

## Abstract

Clinical research has shown that chronic antipsychotic drug (APD) treatment further decreases cortical gray matter and hippocampus volume, and increases striatal and ventricular volume in patients with schizophrenia. D2-like receptor blockade is necessary for clinical efficacy of the drugs, and may be responsible for inducing these volume changes. However, the role of other D2-like receptors, such as D3, remains unclear. Following our previous work, we undertook a longitudinal study to examine the effects of chronic (9-week) typical (haloperidol (HAL)) and atypical (clozapine (CLZ)) APDs on the neuroanatomy of wild-type (WT) and dopamine D3-knockout (D3KO) mice using magnetic resonance imaging (MRI) and histological assessments in a sub-region of the anterior cingulate cortex (the prelimbic [PL] area) and striatum. D3KO mice had larger striatal volume prior to APD administration, coupled with increased glial and neuronal cell density. Chronic HAL administration increased striatal volume in both WT and D3KO mice, and reduced PL area volume in D3KO mice both at trend level. CLZ increased volume of the PL area of WT mice at trend level, but decreased D3KO PL area glial cell density. Both typical and atypical APD administration induced neuroanatomical remodeling of regions rich in D3 receptor expression, and typically altered in schizophrenia. Our findings provide novel insights on the role of D3 receptors in structural changes observed following APD administration in clinical populations.

## Introduction

Over six decades following antipsychotic drug discovery, efforts continue to better understand the mechanisms of action of antipsychotic drugs (APD). Clinical efficacy in the reduction of psychotic symptoms, such as hallucinations, delusions, and disorganized thought, as well as motor side effects have been associated with blockade of D2-like dopamine receptors. The D3 receptor^1,2^, also a D2-like receptor, has both pre- and post-synaptic localization in regions consistently associated with alteration in schizophrenia, such as the limbic cortex, Islands of Calleja, striatum, prefrontal cortex, striatum, and hippocampus^3–7^. Further, the D3 receptors are involved in cognitive, social, and motor functions^3^, many of which are impaired in individuals with schizophrenia. Their preferential blockade with D3 receptor antagonist, S33084, has been associated with pro-cognitive effects in rats^4^. The D3 dopamine receptors are attractive targets for therapeutic intervention in neuropsychiatric disorders in which the dopamine system is dysregulated, such as schizophrenia; this is due to their influence on dopamine release and function, location, and positive effects on cognition^5^.

It is well established that APDs affect neurochemistry, however there is indication that they induce structural and circuit remodeling. Longitudinal magnetic resonance imaging (MRI) studies on the neuroanatomy of individuals with schizophrenia suggest that chronic typical APD treatment - like haloperidol (HAL) - reduces gray matter volume and thickness of the frontal cortices^6–8^. Striatal volume has been shown to increase following chronic typical APD treatment, but to decrease when patients are switched from typical APDs to clozapine (CLZ), an atypical drug used for treatment resistant patients^9^. This may be due to differences in receptor binding and specificity^10^. Importantly, many of these APD effects are confounded by illness severity, duration, and chronicity, as well as the use of multiple medications in treatment. Further, alterations in these regions may exist prior to APD administration as many of these regions are known to be altered in drug naive schizophrenia patients^8,11^.

Rodent models offer an effective way to investigate the impact of APD administration on brain structure, affording precise control over drug exposure, without confounds of illness severity, duration, and other drugs. Both longitudinal and cross-sectional preclinical studies investigating chronic haloperidol or olanzapine (an atypical APD) exposure in both rodents and non-human primates have observed decreased total brain, frontal and parietal cortical volume, and increased striatal volume^12–16^ Furthermore, alterations in shape of the hippocampus have also been reported following chronic HAL or olanzapine treatment^17^. Many of these studies were performed at lower image resolution, which may limit the ability to detect more subtle changes. Furthermore, the role of the D3 receptor in mediating volume changes has never been investigated in previous work.

In a previous study from our group, we investigated the role of the D2 dopamine receptor in modulating APD induced brain volume changes by chronically treating D2 knockout (D2KO) mice and WT littermates with typical and atypical APDs, and found that the D2 receptor played a critical role^18^. However, little is known about the role of the D3 dopamine receptor. Thus, we undertook a longitudinal MRI study with post-mortem histology to investigate the effects of chronic exposure to HALor CLZ on both wild-type and D3 knockout (D3KO) mice in order to characterize the role of the D3 dopamine receptor in brain remodeling induced by chronic APD treatment. As in our previous study, HAL was chosen as it is the most commonly used typical APD, and has been shown to induce structural changes in both clinical and preclinical studies. CLZ was chosen as it is an atypical drug used for treatment resistant patients; it can have severe metabolic side effects, however its effects on brain structure remain unclear. We have chosen to focus our baseline analyses or regions in which the D3 receptor is highly expressed (that could be resolved through our atlas-based segmentation) and in which we later conducted our longitudinal analyses, such as the striatum, nucleus accumbens, globus pallidus, prelimbic area, hippocampus, and hypothalamus^19,20^. We focused our longitudinal analyses on total brain volume (TBV), anterior cingulate cortex (ACC; prelimbic [PL] area specifically), striatum (STR), and hippocampus (HP) following chronic APD administration as these regions have been consistently associated with structural remodeling in both clinical and preclinical studies^6,12,21^. Furthermore, these areas are implicated in the pathology of schizophrenia, and do receive dopaminergic innervation. Finally, these are the same ROIs used in our previous work, which allows for some comparison between the effects of chronic APD on D2KO and D3KO mice^18^. A better understanding of how the dopaminergic system influences structural brain remodeling due to APD administration would aid in the understanding of APD action on the brain, and in the refinement of treatments for schizophrenia.

## Results

### Deletion of D3 dopamine receptor causes subtle anatomical alterations in the mouse brain

**Figure 1:**
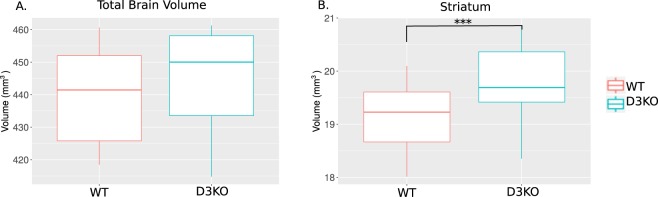
Brain volume differences due to D3 dopamine receptor knockdown. (**A**) Total brain volume was no different between WT and D3KO mice. (**B**) D3KO mice had significantly larger striatal volume following Bonferroni correction (p = 0.0005***). Volume differences are displayed in box plots where the midline represents the median, the box represents the first and third quartiles, and the vertical lines represent the end range of the data.

We observed no significant differences in total brain volume between D3KO and WT mice (p > 0.008, Hedge’s g* = 0.308). We did observe a significantly larger STR in D3KO mice (t = 3.863, p = 0.0005; Hedge’s g* = 1.35707) compared to WT littermates (following Bonferroni correction) (Fig. 1A,B). Other regions rich in D3 receptor expression were not significantly different between WT and D3KO mice including the ACC (PL area specifically) (Hedge’s g* = 0.497), hippocampus (Hedge’s g* = 0.007), nucleus accumbens (Hedge’s g* = −0.027), hypothalamus (Hedge’s g* = −0.234) or globus pallidus (Hedge’s g* = −0.5164) (p > 0.007) (Supplementary Fig. 1A–E).

### Regional volume changes induced by typical and atypical antipsychotics

Multiple comparisons for our 4 ROIs were corrected with for with Bonferroni correction (p < 0.0125).

#### Total brain volume

**Figure 2:**
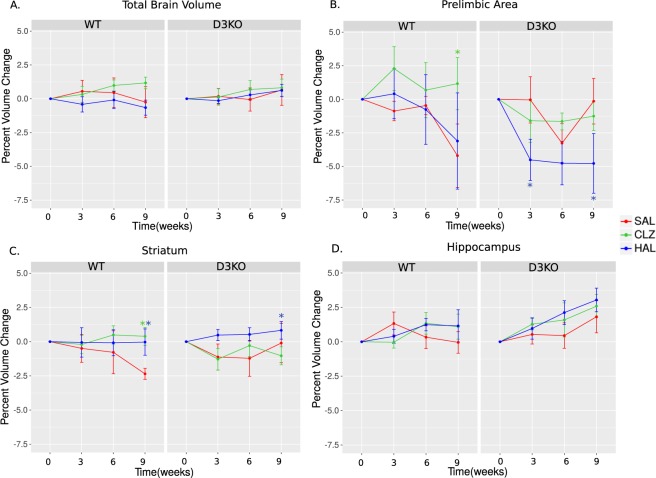
Differences between WT and D3KO mice in response to 9 weeks of APD treatment. All graphs represent percent volume change over time per group. Curves for treatment group represented as follows: saline (SAL in red), haloperidol (HAL in blue) and clozapine (CLZ in green) for D3KO mice and WT littermates. (**A**) Total brain volume was not significantly affected by either HAL and CLZ relative to SAL in either WT or D3KO mice. (**B**) Prelimbic area volume was significantly increased in WT mice for CLZ treated relative to SAL mice (t = −2.335, p = 0.021; green asterix). Volume was reduced in D3KO mice at a trend level at 3 weeks (t = −2.098, p = 0.038) and significant at 6 weeks of treatment relative to SAL (this was not observed in WT mice; t = −2.544, p = 0.012, blue asterix). (**C**) HAL increased striatal volume in WT and D3KO mice relative to SAL (t = 2.623, p = 0.010; blue asterix). CLZ increased STR volume in WT mice relative to SAL (t = −2.004, p = 0.048; green asterix). (**D**) Hippocampal volume changes were not differentially affected by HAL or CLZ relative to SAL for either WT or D3KO mice. All significant three way interactions were decomposed by testing WT and D3KO mice separately, and Bonferroni corrected (corrected p < 0.025).

Analysis of TBV over 9 weeks of treatment revealed no statistically significant differences due to either genotype or treatment (Fig. 2A).

#### Prelimbic area sub-region of anterior cingulate cortex

In the PL region of the ACC we observed a trending genotype × treatment × time interaction for CLZ vs. SAL following 9 weeks of treatment (t = −2.335, p = 0.021) wherein CLZ treatment caused greater reductions in PL volume of D3KO mice relative to SAL, but not HAL. Further, we observed a trending genotype × treatment × time interaction for HAL vs. SAL following 3 (t = −2.098, p = 0.038) weeks and a significant interaction following 9 weeks of treatment (t = −2.544, p = 0.012), wherein HAL decreased PL area volume relative to SAL, but not CLZ, to a greater degree in D3KO mice than WT (Fig. 2B).

To decompose the 3-way interactions observed above, WT and D3KO mice were analyzed separately. For just WT mice, a treatment × time interaction for CLZ vs. SAL was trend level (following Bonferroni correction, p < 0.025) at 3 (t = 2.150, p = 0.037) and 9 weeks (t = 2.206, p = 0.033). For D3KO mice analyzed separately, we observed a significant treatment × time interaction for HAL vs. SAL at 9 weeks (t = −2.695, p = 0.010). These results confirm observations made in the full model.

#### Striatum

Analysis of STR volumes over time revealed a trending genotype × treatment × time interaction following 9 weeks of treatment, for CLZ vs. SAL treatment (t = −2.004, p = 0.048), wherein CLZ treatment seemed to increase STR volume in the WT mice, relative to SAL, but not in the D3KO mice. Furthermore, there was a significant treatment × time interaction for HAL vs. SAL following 9 weeks (t = 2.623, p = 0.010) of treatment and trending at 3 weeks (t = 1.714, p = 0.08), suggesting that HAL increased STR volume in both WT and D3KO mice over time (Fig. 2C).

For WT mice analyzed alone (without D3KO mice), we observed a trend level (following Bonferroni correction, p < 0.025) treatment × time interaction for HAL vs. SAL at 9 weeks of treatment (t = 2.164, p = 0.036). For D3KO mice analyzed separately, we observed a trend level treatment × time interaction for CLZ vs. SAL at 3 (t = −1.782, p = 0.082) and 9 weeks of treatment (t = −2.225, p = 0.0317).

#### Hippocampus

Similarly to TBV, no significant differences were observed in HP volume over time (Fig. 2D).

### Stereological assessment of glial and neuronal population densities in the prelimbic area and striatum of D3KO mice and WT littermates

**Figure 3:**
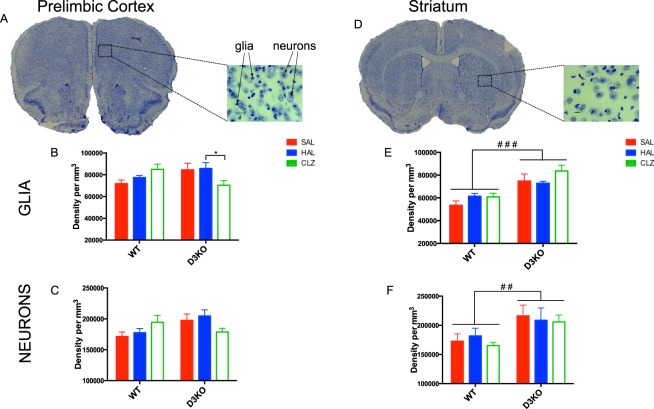
Chronic typical and atypical APD treatment differentially affects neuronal and glial cell density in the prelimbic area and striatum of WT and D3KO mice. (**A**) Representative Nissl stained coronal section at AP = + 1.98 from bregma taken from a WT-SAL treated mouse (Figure 14 from page 40 of the Franklin & Paxinos Mouse Brain Atlas 3rd edition^50^). Zoom in of prelimbic area of the anterior cingulate cortex to show differences between glial and neuronal cells. Glial and neuronal cell density of PL for each treatment group saline (SAL in red), haloperidol (HAL in blue) and clozapine (CLZ in green) for D3KO mice and WT littermates. (**B**) Glial cell density is significantly greater following HAL compared to CLZ treatment in D3KO mice (+18%, p = 0.048). (**C**) D3KO mice tend to have higher neuronal density following either SAL or HAL treatment (p = 0.09). (**D**) Representative Nissl stained coronal section at AP = +0.38 from bregman taken from a WT-SAL treated mouse (Figure 28 from page 55 of the Franklin & Paxinos Mouse Brain Atlas 3rd edition^50^). Zoom in of STR to show differences between glial and neuronal cells. (**E**) D3KO mice had significantly greater glial cell density (+20.3%; p < 0.00001). (**F**) D3KO mice had significantly greater neuronal cell density (+12.5%; p < 0.007).

For glial cell density in the PL area of the ACC we found a significant treatment by genotype interaction (F(2,27) = 5.66, p = 0.009). Post-hoc comparisons revealed that HAL increased glial cell density relative to CLZ (+18%, p = 0.048) **(**Fig. 3B). There was also a significant treatment by genotype interaction for neuronal density measures (F(2,27) = 4.16, p = 0.027), with a tendency for D3KO mice to have a greater neuronal density (+15%) than WT mice following either SAL or HAL treatment (p = 0.09) (Fig. 3C).

For STR measures, D3KO mice had significantly greater glial cell (+20.3%; F(1,18) = 29.68, p = 0.00001; Fig. 3E**)** and neuronal cell density (+12.5%; F(1,18) = 9.120, p = 0.007; Fig. 3F**)**.

## Discussion

The D3 dopamine receptor is highly expressed in the limbic system, and is of particular interest as a potential mediator of aberrant dopaminergic neurotransmission. It plays a critical role in cellular and synaptic plasticity throughout brain development. Although the D2 dopamine receptor is more highly expressed in the brain, and is the main target of APDs, careful investigation of APD affinity through PET imaging demonstrates that both D2 and D3 receptor binding is necessary for reaching therapeutic effects^22^.

Although D3 receptor function is important, its role in modulating brain morphology in development as well as due to chronic blockade via APD treatment had not been investigated. The availability of transgenic dopamine D3KO mice allow us to study interactions between the receptor and brain anatomy. This is the first study to investigate neuroanatomical alterations due to deletion of the D3 receptor, and how this deletion interacts with chronic APD treatment.

Firstly, we observed an increase of striatal volume in D3KO mice, independent of total brain volume changes. The striatum is a region in which the D3 receptor would typically be expressed, thus it is possible that in its absence disrupts certain developmental processes, creating an increase in cell proliferation or density. However, the limited structural alterations we observe in D3KO mice may be due to the relatively sparse and heterogeneous expression of the receptor throughout the brain^19,23^. Striatal volume increase was accompanied by an overall increase in glial and neuronal cell density in all three treatment groups. In our previous study, investigating the effects of chronic APDs on D2KO mice and WT littermates, we observed a striking increase in striatal volume, and a decrease in glial cell density in the STR of D2KO mice following HAL and CLZ treatment^18^. The fact that we do not observe a similar effect in this study suggests that D3 receptor removal prevents APDs from altering glial density. This is of interest since D3 receptors are more densely expressed in glia than neurons, suggesting that the D3 receptor may play a critical role in modulating APD effects on glial cells^24^.

Next, we observed that chronic HAL administration increased striatal volume in both WT and D3KO mice. Typical APD treatment has consistently been shown to increase striatal volume in both clinical and preclinical literature, using both MRI and post-mortem investigations^7,9,12,13^. Thus, it is unsurprising that HAL treatment increased striatal volume in WT mice; the increase observed in D3KO mice is of interest, suggesting that the D3 dopamine receptor is not necessary for this structural remodeling. These changes may therefore be more D2 receptor dependent, as in our previous study, we did observe trending striatal volume increases following chronic HAL treatment in WT, but not D2KO mice. These volumetric findings were not backed up by histological findings, however these volume differences might be more subtle than the bulk increases in glial and neuronal cell density due to D3 receptor knock down.

Finally, we observed that CLZ increased volume of the ACC subregion, the PL area, compared to SAL treated WT mice. In D3KO mice, HAL treatment reduced PL area volume relative to SAL treated mice. CLZ treatment seemed to increase PL area volume relative to HAL treatment (although not statistically significant), coupled with a CLZ-induced decrease in PL area glial cell density. Previous work from both rodent and non-human primate studies has shown that chronic HAL and olanzapine treatment actually increases PL area glial cell density (both astrocytes and microglia), coupled with decreases in volume^12,21,25^. Therefore, these studies suggest that the volume decreases observed may be due to a loss of dendritic arborization, and not necessarily a loss in cell number. We also do not observe HAL induced changes to neuronal or glial cell density in the PL, which is surprising given that in our previous study HAL did increase glial cell density in WT mice mice only^18^. It is possible that if we investigated astrocyte- and microglia- cell density separately we would have seen similar effects. Previous work has shown that chronic and acute CLZ treatment increases c-fos mRNA expression in the rat prelimbic area, whereas HAL does not^26^. We were surprised to observe a decrease in PL area volume due to HAL treatment only in the D3KO mice, as both clinical and preclinical studies have shown that chronic typical APD treatment reduces frontal cortex volume^6,12^. HAL treatment does also decrease volume of PL area in WT mice, however so does SAL treatment. Previous work, using similar dosing and drug administration route (10 mg/kg CLZ daily i.p. injections) also observed alterations to expression and epigenetic function of histone deacetylase 2 in frontal cortex, via the 5-HT2A receptors, possibly suggesting an alternative route to the dopaminergic system for explaining volume changes due to CLZ^27^.

It is important to consider this study in light of some limitations. We observe interesting effects, however many are at a subthreshold level, possibly due to our modest sample size of 6 mice of mixed sex per group. Furthermore, the mice were fairly old at the start of treatment and the ages were not perfectly matched between groups (range from 20–28 weeks). Therefore, it is possible that APD treatment may have affected mice differently based on their age, even though all mice were adults, and fully developed at the time of treatment initiation. In future work, it would be important to determine whether an inducible D3KO mouse model (occurring in adulthood), would result in a similar response to chronic APD administration, to rule out any compensatory mechanisms. We do observe subtle volume changes at baseline in the D3KO mice, so it is possible that certain neurodevelopmental compensations are occurring, however previous work has shown that these mice do develop normally, achieve fertility, and have only subtle behavioural alterations in comparison to their WT littermates, such as transient locomotor hyperactivity in a novel environment^28,29^.

Additionally, as discussed in our previous paper^18^, daily intraperitoneal injections may have affected D2-like receptor occupancy, and stress levels differently than a constant infusion via osmotic minipumps would have. This may help explain the decrease in ACC’s PL area volume of WT mice due to SAL. However, the majority of our results are consistent with those observed in other clinical and preclinical findings investigating interactions between chronic APD treatment and brain anatomy. Based on previous work by Kapur^30^, in order to achieve 50% D2 receptor occupancy in male rats, required doses are as follows: HAL, 0.04–0.08 mg/kg subcutaneous and clozapine, 5–15 mg/kg subcutaneous, however this is based on a single injection. This is slightly below the ~70% D2 receptor occupancy typically achieved in clinical settings, however it would still suggest that our HAL dose may be achieving slightly higher D2 occupancy than what is clinically comparable. PET studies in humans observed that high levels of D2 occupancy are reach shortly after drug administration, but that levels drop after 12 hours of injection^31^. Preclinical studies in rats shown that daily subcutaneous HAL injections at 1 mg/kg reach 94% D2 occupancy at their peak, but only 17% at the trough, suggesting that APD effects may be achievable without sustained D2 occupancy^32^; similar observations were noted for daily i.p. injections^33^. Conversely, when similar doses were administered via osmotic minipump D2 receptor occupancies were stable throughout the day, but perhaps lower than clinically therapeutic levels^32^. It is possible that given the stability of D2 occupancy achieve via osmotic minipump use, this method is more clinically comparable, however it is unclear whether constant D2-receptor blockade is necessary for clinical occupancy. In fact, Remington and Kapur suggest that more intermittent blockade could be beneficial^34^.

Finally, although HAL is the most classically used typical APD, and CLZ is of interest for treatment resistant individuals, it would have been interesting to investigate an APD that targets D2 and D3 receptors more specifically, such as amisulpride^35^. It is possible that this choice of APD would have induced brain volume changes in the WT, but not D3KO mice. Obviously the D3 dopamine receptor is not the main target of APDs, although it is part of the D2-like family. This has been investigated to some degree in human studies. Girgis and colleagues found that the risperidone, an atypical APD, does bind to the D3 receptor in regions like the substantia nigra and the ventral tegmental area^36^. Conversely, Mizrahi and colleagues found that patients treated with atypical APDs did not seem to have D3 receptor occupancy due to treatment, however they did have an upregulation of D3 receptor following short-term treatment^37^. In future work it would be interesting to investigate how the degree of D3 receptor binding of different drugs affects brain morphology.

Additionally, an investigation of other regions expressing the D3 receptors would be of interest. We did not observe baseline differences in a D3 rich region like the nucleus accumbens, however it is possible that histological investigation would show more subtle alterations. Furthermore, chronic clozapine, but not haloperidol treatment in rats was shown to increase c-fos mRNA in both the prelimbic and infralimbic cortices^26^. Furthermore, the D3 receptor has recently been shown to be expressed in the infralimbic cortex, as well as other regions of the medial prefrontal cortex^38,39^ thus a more thorough investigation of these brain regions could be insightful in our understanding of the D3 receptors’ involvement in structural remodeling.

In future work, it would also be interesting to investigate volume changes at the voxel-level; this was not performed in the current study, as we were not sufficiently powered with modest group sizes and many treatment-genotype subgroups of mixed sex. A similar study with larger groups of male and female mice would allow for thorough investigation of sex differences; this would be appropriate and of interest given known sex differences in symptomatology in schizophrenia, and in response to APD treatment. Furthermore, a thorough comparison of treatment with continuous drug infusion using osmotic mini-pumps or daily single injections would further our understanding of the relationship between brain volume changes and consistent vs. intermittent blockade of D2-like receptor systems.

In conclusion, we present evidence for the role of the D3 dopamine receptor in modulating plasticity of the striatum, both at the volume and cellular level. Further, we observe that HAL significalty impacts PL area and STR volume in both WT and D3KO mice, and that the lack of D3 receptor prevents striatal glial cells from being affected by APD treatment. The D3 receptor may offer new therapeutic targets for drug treatments, therefore a better understanding of how APDs interact with the D3 dopamine receptor is important for furthering our understanding of how APD treatment affects the brain.

## Materials and Methods

### Animals: housing and breeding

D3KO mice engineered in the 129S4/SV strain by Sarah Fuchs’s group^28^ were purchased from Jackson laboratories (B6;129S4-*Drd3*^*tm1Dac*^/J stock#002425), and maintained by backcross-breeding with C57BL/6 J mice. F1 heterozygote D3KO (+/−) male and female mice (with >10 backcross generations)^40^ were bred in order to obtain both homozygous D3KO (−/−) and wild type (WT; +/+) littermates. Upon sexing and weaning, tail snips were collected from each mouse and used for genotyping to assess the presence of the Drd3 gene (homozygotes, heterozygotes, or wild-type) using polymerase chain reaction (PCR). D3KO (+/−) heterozygote offsprings were discarded and mice from the same litter and from the same sex with mixed WT and KO genotypes were housed 3–4 per cage and kept as it during treatment and imaging. Animal housing, breeding, and care were conducted in accordance to the Canadian Council on Animal Care guidelines (CCAC; http://ccac.ca/en_/standards/guidelines) and the Animal Care Committee of the Douglas Institute. All experiments were performed according to CCAC and the Douglas ACC under protocol 2008–5570 (renewed 06/01/2016). The mice were kept under standard conditions at 22 ± 1 °C, 60% relative humidity, and 12-h light-dark cycle with food and water available *ad libitum*.

### Experimental design

Male and female D3KO and WT mice were randomly assigned to one of three treatment groups: NaCl 0.9% (saline (SAL)); haloperidol (HAL; 1 mg/kg); or clozapine (CLZ; 5 mg/kg; Sigma-Aldrich, St Louis, USA). Each treatment group had 6 mice (3 males/3 females), with mean age 23 ± 4 and 24 ± 4 for the WT and D3KO saline (SAL) group, 21 ± 5 and 20 ± 5 for the WT and D3KO haloperidol (HAL) group, and 28 ± 4 for the WT and D3KO clozapine (CLZ) groups. Drugs were administered daily via intraperitoneal (i.p.) injections for 9 weeks, and mice were scanned at 0, 3, 6, and 9 weeks of treatment. Following the last scan, brains were fixed by paraformaldehyde (PFA) via intracardiac perfusion, and were extracted for histological assessment of neuronal and glial cell populations (details in Sections 2.6 & 2.7).

### Drug preparation

HAL was prepared by dilution of injectable human doses (50 mg/mL) obtained from the Douglas Mental Health University Institute, Montreal, QC, Canada. A fresh solution was prepared by dilution to a final 1 mg/kg concentration in sterile NaCl 0.9% solution every two days. A CLZ stock solution (Sigma-Aldrich, St Louis, USA) was prepared by dissolving the powder in 0.1 M HCl and stored at −80 °C (500 μl aliquots at a concentration of 10 mg/ml). Every 3 days, an aliquot was diluted in NaCl 0.9% at 5 mg/kg, and pH was adjusted to physiological levels (7.4) with NaOH. As in our previous work^18^, doses of HAL and CLZ were chosen to being 2–3 times higher than an acute dose producing inhibition of locomotion in C57Bl/6 mice^41^, and were coherent with other animal studies in rats^12,15,42^.

### MRI image acquisition

Animals were scanned *in vivo* (1–3% isofluorane) using a 7 T Bruker BioSpec small animal scanner (Bruker BioSpin Corporation, Billerica, MA). A Bruker 112–086 mm circularly polarized resonator and mouse head surface coil were used as transmit and receive antennas, respectively. A standard Bruker 3D-TrueFISP sequence was used to collected 140 μm isotropic voxel resolution images with the following parameters: FOV 1.80 × 1.80 × 0.90 cm, 128 × 128 × 64 matrix, TE/TR 2.6/5.2 ms, NEX 2, TA 3 m 24 s. Eight RF phase angles were used (180, 0, 90, 270, 45, 225, 135, and 315 degrees) to remove banding artifacts. Respiration was monitored and maintained between 30–50 breaths per minute (1025-IBP-50 Small Animal Monitoring Gating System; SA instruments www.i4sa.com). Body temperature was maintained by blowing warm air on the animal (40 minute scan timel. Final images used for analysis were root mean square (RMS) averages of the 8 acquisitions. Slight motion during scans was corrected for following the acquisitions using rigid registration using FLIRT in FSL tools^43,44^.

### MRI Image Analysis: automatic segmentation using MAGeTBrain

The automatic segmentation tool, MAGeTBrain was used to generate regional brain volumes^45^. The Dorr atlas was used to label 111 brain regions, including the striatum (STR) and hippocampus (HP), two of our regions of interest (ROIs)^46^. This atlas does not label subregions of the frontal cortex, so we also performed the segmentation using the Dorr-Steadman-Ullman (DSU) atlas (289 labeled regions) to obtain volume of the anterior cingulate cortex sub-region, the prelimbic area (PL), as it was one of our ROIs^46–48^. We performed linear and non-linear registration using ANTS^49^ to align the high-resolution atlas to 25 template subjects, stratified across the data. All other subjects were then warped to the 25 templates, yielding 25 candidate segmentations per subject; a voxel majority-voting procedure was used to select the final voxel label. Quality control was performed on all outputs by visual inspection.

### Brain tissue processing

Immediately after the last scan, mice were transcardially perfused with 4% paraformaldehyde (PFA) in phosphate buffered saline solution (following pentobarbital overdose) Extracted brains were post-fixed in PFA overnight at 4 °C. A vibratome was used to slice 30 µm thick coronal sections of the PL area and STR (Leica, Freiburg, Germany). One in every four sections (per ROIs, per animal) were mounted on Superfrost + slides (Fisher Scientific, Pittsburg, USA), allowed to dry overnight, and Nissl-stained with cresyl violet acetate solution according to standard methods. Not all mice were used for Nissl-staining, as tissues were used to test other markers (data not shown), yielding final groups as follows (for the PL area: WT-SAL n = 6, WT-HAL n = 4, WT-CLZ n = 6, D3KO-SAL n = 6, D3KO-HAL n = 6, D3KO-CLZ n = 6; for the STR: WT-SAL n = 4, WT-HAL n = 4, WT-CLZ n = 4, D3KO-SAL n = 6, D3KO-HAL n = 6, D3KO-CLZ n = 4).

### Stereological quantification of neuronal and glial densities

Stereological quantification was performed as previously described^18^. Briefly, systematic random sampling was performed using an optical fractionator probe (Stereo Investigator 11.01.2, MBF Bioscience, Williston, VT) with a section sampling fraction of 1/4, on a Zeiss Axio Imager M2 microscope equipped with a motorized stage and Axiocam MRc camera. Borders for ROIs (PL area (bregma: +2.33 to +1.69) and STR (bregma: +1.33 to +0.38)) were drawn at 2.5×, referencing the Franklin and Paxinos atlas^50^. Neurons and glia were counted using StereoInvestigator (version 11.01.2, MBF Bioscience, Williston, VT) at 63× magnification (Plan-apochromat objective, 1.4 numerical aperture), and identified based on morphological differences (for neurons: presence of a visible nucleolus and cytoplasm, for glia, very dark round shape^51,52^. Counting parameters were as follows: counting frame = 60 μm^2^ sampling grids = 260 μm^2^ for PL area and 300 μm^2^ for the STR. In calibration studies, these parameters gave Gundersen (m = 1) coefficient of error (CE) < 0.07 for neurons and glial cells for both regions. Section thickness was measured within each counting frame, with a mean thickness of 14 μm. Dissector height was set at 8 μm (3 μm guard zone distance). The volume of the area was determined using the Cavalieri Estimator probe (100 μm grid spacing; 30 μm thick sections). Densities of cell populations for each ROI were calculated by dividing their respective population estimates obtained by the optical fractionator workflow, by the Cavalieri volume estimation. Mean Gundersen for neurons and glial cells were respectively 0.06, 0.08 in the left and right PL area and 0.05, 0.08 in the left and right STR.

### Statistical analysis

#### Baseline genotype differences

All statistics using MRI data were performed using RMINC/1.4.3.4 (https://wiki.mouseimaging.ca/display/MICePub/RMINC) in the R statistical environment (www.r-project.org).

A linear model was used to test baseline genotype differences in structure volume between D3KO mice (n = 18) and WT littermates (n = 18) using sex and age as covariates. Regions known to express the D3 receptor in high amounts, such as the striatum, nucleus accumbens, hippocampus, prefrontal cortex, and hypothalamus were selected. A Bonferroni correction was applied to correct for multiple comparisons (p = 0.05/7 = 0.007).

#### Longitudinal assessment of APD on volume

Differences in slope for each ROI (PL area, STR, HP, and TBV) were tested using a linear mixed effects model (LMEM) testing for genotype-by-treatment-by-time interactions, with sex, age at baseline, and TBV (except when analyzing TBV) as covariates, and subject as the random intercept^53^. SAL was used as the treatment reference, which allowed for comparison of HAL and CLZ to SAL). In order to statistically decompose the three-way interactions (when significant), WT and D3KO mice were analyzed separately using a LMEM testing for treatment-by-time interactions, covarying for sex and age at baseline. A Bonferroni correction was applied to the linear mixed effects models performed on the 4 ROIs (p = 0.05/4 = 0.0125) as the significance threshold.A Bonferroni correction was also applied to the post-hoc testing of each genotype separately with both SAL and HAL as reference variables, with p = 0.05/4 = 0.0125 set as the significance threshold.

## Supplementary information

Supplementary Informations
