# Supplementary material for: Role of D3 dopamine receptors in modulating neuroanatomical changes in response to antipsychotic administration
Source: Sci Rep. 2019 May 24;9:7850. doi: 10.1038/s41598-019-43955-4 (PMC6534671; doi:10.1038/s41598-019-43955-4)
Supplement: Supplementary file 1 — Supplementary Informations [file 41598_2019_43955_MOESM1_ESM.pdf]

Role of D3 dopamine receptors in modulating neuroanatomical changes in response to antipsychotic administration

Elisa Guma[1,2], MSc, Jill Rocchetti PhD[1], Gabriel A. Devenyi PhD[2,4], Arnaud Tanti PhD[3], Axel P. Mathieu[2], PhD, Jason P. Lerch PhD[6,7,8,9], Guillaume Elgbeili MSc[4], Blandine Courcot PhD[2], Naguib Mechawar[3,4], PhD, M Mallar Chakravarty PhD[1,2,4,5], Bruno Giros[1,4,10]

[1] Integrated Program in Neuroscience, McGill University, Montreal, Quebec, H3A2B4, Canada

[2] Cerebral Imaging Center, Douglas Mental Health University Institute, Montreal, Quebec, H3H1R3, Canada

[3] McGill Group for Suicide Studies, Department of Psychiatry, McGill University, Douglas Mental Health University Institute, Montreal, QC, Canada

[4] Department of Psychiatry, McGill University, Montreal Quebec H3A1A1, Canada

[5] Department of Biological and Biomedical Engineering, McGill University, Montreal, Quebec, H3A2B4, Canada

[6] Mouse Imaging Centre, The Hospital for Sick Children, Toronto, Ontario, M5T3H7, Canada.

[7] Department of Neurosciences and Mental Health, The Hospital for Sick Children, Toronto, Ontario, M5G1X8, Canada.

[8] Department of Medical Biophysics, University of Toronto, Toronto, Ontario M5G1L7, Canada

[9] Wellcome Centre for Integrative Neuroimaging, University of Oxford.

[10] Sorbonne University, Neuroscience Paris Seine, CNRS UMR 8246, INSERM U 1130, UPMC Univ Paris 06, UM119, 75005, Paris, France

° Corresponding author: bruno.giros@mcgill.ca

SUPPLEMENTARY MATERIAL

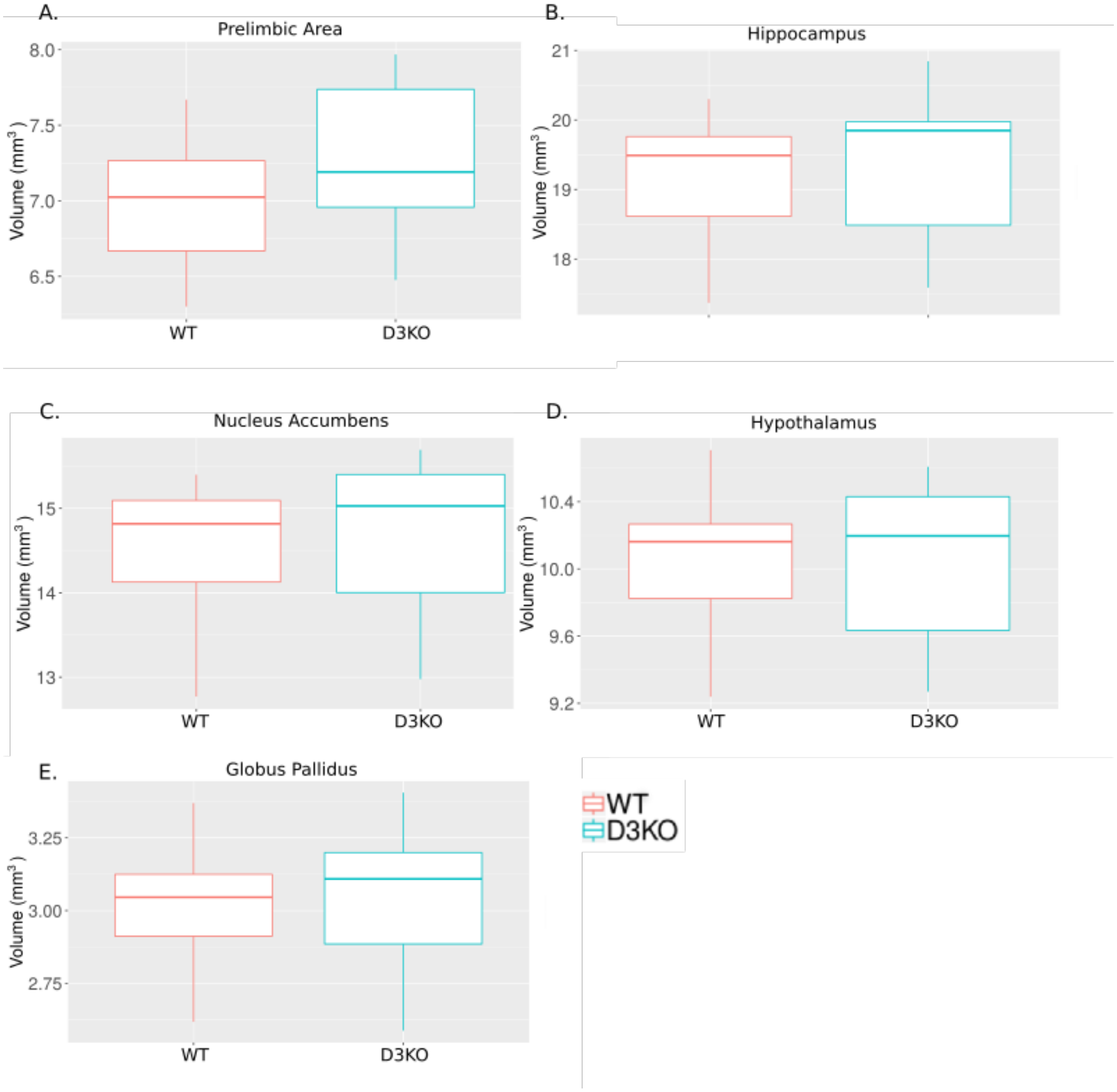

Supplementary Figure 1. Volume comparison between WT and D3KO mice in regions known to express the D3 receptor. Prelimbic area **(A)**, hippocampus **(B)**, nucleus accumbens **(C)**, hypothalamus **(D)**, and globus pallidus **(E)** were not significantly different following Bonferroni correction ($p<0.007$). Volume differences are displayed in box plots where the midline represents the median, the box represents the first and third quartiles, and the vertical lines represent the end range of the data.

Tables reporting stereological data including mean cell count, SD, CV and Gudernsen co-efficients for both neurons and glia in the prelimbic area and striatum as follows.

Table S1. Summary of stereological data for prelimbic area neurons

Group	Estimated Population using Mean Section Thickness	Cavallieri Estimated Volume (µm³)	Density	Gunderson Coefficient Error
WT-SAL	61601.18	360600000.00	171246.20	0.05
WT-HAL	66854.41	382714285.71	174063.98	0.05
WT-CLZ	65630.57	372100000.00	176749.78	0.05
KO-SAL	75368.73	382200000.00	197306.42	0.05
KO-HAL	81610.20	399120000.00	204699.16	0.05
KO-CLZ	74176.25	416800000.00	178658.36	0.05

Table S2. Summary of stereological data for prelimbic area glial cells

Group	Estimated Population using Mean Section Thickness	Cavallieri Estimated Volume (μm³)	Density	Gunderson Coefficient Error
WT-SAL	25833.52	360600000.00	71759.18	0.08
WT-HAL	29345.60	382714285.71	76669.71	0.08
WT-CLZ	28492.90	372100000.00	75906.58	0.07
KO-SAL	32348.47	382200000.00	84506.98	0.07
KO-HAL	34387.63	399480000.00	85859.63	0.07
KO-CLZ	29214.47	416800000.00	70335.52	0.07

Table S3. Summary of stereological data for striatum neurons

	Estimated Population using Mean Section Thickness	Cavallieri Estimated Volume (μm³)	Density	Gunderson Coefficient Error
WT-SAL	128889.48	787230000.00	172804.03	0.06
WT-HAL	113634.01	631500000.00	194040.77	0.07
WT-CLZ	118605.38	713520000.00	186217.79	0.06
KO-SAL	132141.22	614850000.00	216273.42	0.05
KO-HAL	132141.22	614850000.00	216273.42	0.05
KO-CLZ	132795.80	624360000.00	197224.96	0.07

Table S4. Summary of stereological data for striatum glial cells

	Estimated Population using Mean Section Thickness	Cavallieri Estimated Volume (μm³)	Density	Gunderson Coefficient Error
WT-SAL	39808.25	787230000.00	53496.46	0.09
WT-HAL	38094.93	765750000.00	53000.01	0.09
WT-CLZ	34352.20	659190000.00	54816.23	0.09
KO-SAL	43069.51	773310000.00	57198.99	0.08
KO-HAL	32320.13	517710000.00	67476.89	0.10
KO-CLZ	39446.96	657000000.00	66291.18	0.08
